# Early Degenerative Effects of Diabetes Mellitus on Pancreas, Liver, and Kidney in Rats: An Immunohistochemical Study

**DOI:** 10.1155/2012/120645

**Published:** 2012-07-11

**Authors:** Mehmet Haligur, Senay Topsakal, Ozlem Ozmen

**Affiliations:** ^1^Department of Pathology, Faculty of Veterinary Medicine, University of Mehmet Akif Ersoy, 15030 Ortulu Yerleskesi, Burdur, Turkey; ^2^Department of Endocrinology and Metabolism, Faculty of Medicine, University of Pamukkale, 20070 Kinikli, Denizli, Turkey

## Abstract

Liver and kidney commonly affected by diabetes in chronic cases but pathogenetic mechanisms are not fully understood in early stages of the disease. The aim of this study was to investigate the immunohistochemical expression of caspase-3, cyclooxygenase (COX)-1 and-2, calcium sensing receptor (CSR), and hypoxia inducible factor-1**α** (HIF-1**α**) in pancreas, liver, and kidney in streptozotocin (STZ) induced DM. Study group (*n* = 6) were received streptozotocin (50 mg/kg) and control group (*n* = 6) physiologic saline. The blood glucose and ketonuria were measured, and necropsy was performed on them on third, fourth, and fifth days. Immunohistochemistry revealed that marked increase in caspase-3 reaction pancreas, liver, and kidney in the study group than control group. COX-1 slightly increased in these organs in study group compared to controls. Immunohistochemically COX-2 reaction was markedly positive in liver and kidney, but slightly increased in pancreas. The most increased reaction was observed in CRS and all organs were markedly positive. HIF-1**α** expression was also increased but the reaction was more severe in pancreas than liver and kidney. This study indicated that degeneration starts in organs in early stages of the disease and the most effective route for degeneration related to increase of calcium influx and hypoxia upon cells in DM.

## 1. Introduction

Diabetes mellitus is a metabolic disorder that results from a reduction of insulin available for normal function of many cells in the body. In some cases, increased concentrations of glucagon contribute to development of persistent hyperglycemia. In addition to chronic hyperglycemia, DM is characterized by disturbance of carbohydrate, fat, and protein metabolism resulting from defects in insulin secretion, insulin action, or both. The diseases can also be recognized during less overt stages, most usually by the presence of glucose intolerance. The effects of DM include long-term damage, dysfunction, and failure of various organs, especially the eyes, kidneys, livers, hearts, and blood vessels [1].

In the pathogenesis of DM, several factors are responsible for the decreased availability of insulin. Hyperglycemia, and its attendant effects upon cells, underlies the pathogenic lesions of DM [2]. Cellular damages can be demonstrated by numerous markers by immunohistochemistry. For example, caspases are a family of cysteine proteases mainly involved in the apoptotic pathway [3]. Caspase-3 is one of the effector caspases that has been implicated as a key protease cleaving multiple cellular substrates, including components related to DNA repair and regulation, to bring the cell to its demise [4, 5]. Cyclooxygenase enzymes also play an important role at cellular damages. Three different COX enzymes existed, now known as COX-1, COX-2, and COX-3, they are responsible for formation of important biological mediators called prostanoids, including prostaglandins, prostacyclin, and thromboxane. Pharmacological inhibition of COX can provide relief from the symptoms of inflammation and pain [6]. In diabetes, direct evidence that cytoplasmic Ca^2+^ triggers exocytosis of the insulin granules is obtained from experiments using *β* cell of which the plasma membrane is permeabilized in these cells, the membrane potential is dissipate and the cytosolic concentration of small molecules can be controlled [7]. Calcium sensing receptor is a G protein-coupled receptor (GPCR) and regulation of extracellular and intracellular calcium homeostasis related to CSR. It has also been found in a wide variety of organs not involved in systemic calcium homeostasis that it plays important roles in cellular damages [8–10]. Hypoxia-inducible factor-1*α* has central role in degeneration and a transcriptional activator that promotes angiogenesis [11, 12]. HIF-1*α* expression is also induced under normoxic conditions when cells are stimulated with growth factors, inflammatory cytokines, lactate, or prostaglandins [13–15].

DM is a complex disease and causes numerous cellular damages in different organs. A number of pathogenetic advances have been made during the past decade but numerous mechanisms need to be clarified. The pathogenetic mechanisms are likely interactive and linked in DM. For that reason, mechanism of cellular damage is not fully understood. This preliminary study was designed to explore the cellular distribution and the underlying mechanisms of hypoxia and calcium influx in experimental diabetes.

## 2. Material and Methods

Twelve female Sprague-Dawley rats, weighing 125 to 150 g and aged 2 months, were maintained at the Experimental Animal Housing Unit of the University of Akdeniz. They were randomly allocated into 2 groups, as follows: study group that treated with STZ and control group. Both groups were composed of 6 rats and were allowed free access to water and food. Rats were fasted before the STZ injection. A single intraperitoneal injection of 50 mg/kg STZ (Sigma Chemical Co, St. Louis, Mo) dissolved immediately before administration in freshly prepared 50 mmol/L citrate buffer (pH 4.0) that was given on day 0. Control animals received an equivalent volume of physiologic saline. Urine samples were collected at 3rd, 4th, and 5th days. At the third day, two rats form, study group and two rats from the control group were anesthetized with ether before blood and tissue samples were obtained. The blood glucose concentration was measured in blood from jugular vein in the morning from 2 rats in each group before euthanasia, and then necropsy was performed on them after the third day. The MS9 blood counting equipment was used for hematological analysis of the blood drawn in EDTA tubes. Glucose levels were analyzed in serum samples using IDEXX VetTest equipment and reagents. Pancreas, liver, and kidney tissue samples were collected and fixed in 10% buffered formalin. After routine procedure, tissues were blocked in paraffin and cut to 5 *μ*m thickness. Tissue sections were stained with hematoxylin-eosin (HE) and examined microscopically. Afterward, pancreas, liver, and kidney samples were immunostained with caspase-3 (rabbit polyclonal, Cat. no. 250573, Abbiotec-San Diego, USA), COX-1 (Epitope Specific rabbit antibody, Cat. no. RR-10687-P0, Thermo scientific, Fremont, USA), COX-2 (Cat. no: RM-9121-S0, Thermo scientific, Fremont, USA), CSR (Rb pAb to CSR, ab62653-100, Abcam Lot: 433372, Cambridge, UK), and HIF-1*α* (H1*α*67, Sc-53546, Santa Cruz Biotechnology Inc. CA, USA) according to the manufactures' instructions. In this study, avidin-biotin complex peroxidase (ABC-P) method was used for immunohistochemistry. Paraffin blocks were sectioned at 5 *μ*m for immunohistochemical examination, and sections were attached to glass slides coated with poly-L-lysine. The slides were dried overnight at 37°C to optimize adhesion. Sections were deparaffinized through xylene, and tissues were rehydrated in sequentially graduated ethyl alcohol. Slides were incubated in hydrogen peroxide in methanol for 10 min to reduce nonspecific background staining due to endogenous peroxidase. The sections were washed twice, in phosphate buffer solution (PBS). Then, tissues were boiled in 1 : 100 citrate buffer solution for10 min and cooled for 20 min. The cooled tissues were washed four times in PBS prior to application of blocking serum for 5 min. Then, primary antibody was applied; tissues were incubated for 30 min at room temperature. They were rinsed 4 times in PBS, given an application of biotinylated anti-polyvalent antibody and incubated for 10 min at room temperature. After being washed three times in PBS, streptavidin peroxidase was applied and the samples were incubated for 10 min at room temperature, and then rinsed 4 times in PBS. Tissues were further incubated for 20 min at room temperature in a solution of DAB (3, 30 diaminobenzidine) chromogen. After being washed in PBS, tissues were counter stained with Mayer's haematoxylin, washed in water, and coverslips were applied with mounting media. For negative control, primary antibody was not added to the sections.

In order to evaluate the percentage of immunopositive cells, 100 cells calculated in 10 different microscopic high-powered fields of each slide were examined under the 40x objective of a trinocular microscope (Nikon E600) and microphotography apparatus. The count of positive cells one high-power field for each marker was noted and compared with control groups.

In the statistical evaluations, Students *t* test was used. Calculations were made using the SPSS 13.0 program pack. *P* < 0.05 was accepted as statistically significant.

## 3. Results

**Figure 1:**
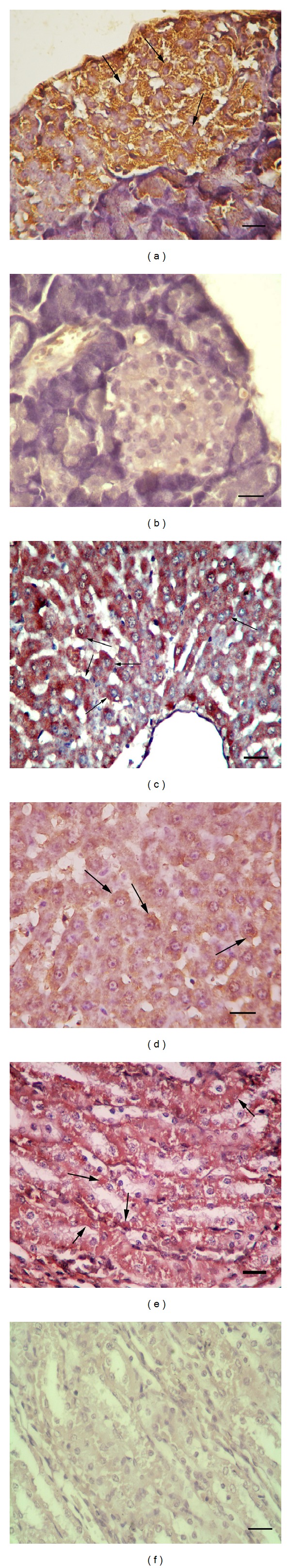
*Caspase-3  reactions*. (a) Immunopositive reaction in Langerhans islets cells (arrows) in study group, Bar = 50 *μ*m; (b) no immunoreaction in control group, Bar = 50 *μ*m; (c) marked reaction in hepatocytes in study group (arrows), Bar = 100 *μ*m. (d) Slight immunoreaction in some hepatocytes (arrows) in control group, Bar = 50 *μ*m; (e) Strong reactions in tubular cells in kidney (arrows), Bar = 100 *μ*m. (f) A few immunopositive cells in kidney in control group, Bar = 100 *μ*m. ABC-P method with Hematoxyline counterstaining were used for all tissue. The right column belongs to study group and left column belongs to control group.

**Figure 2:**
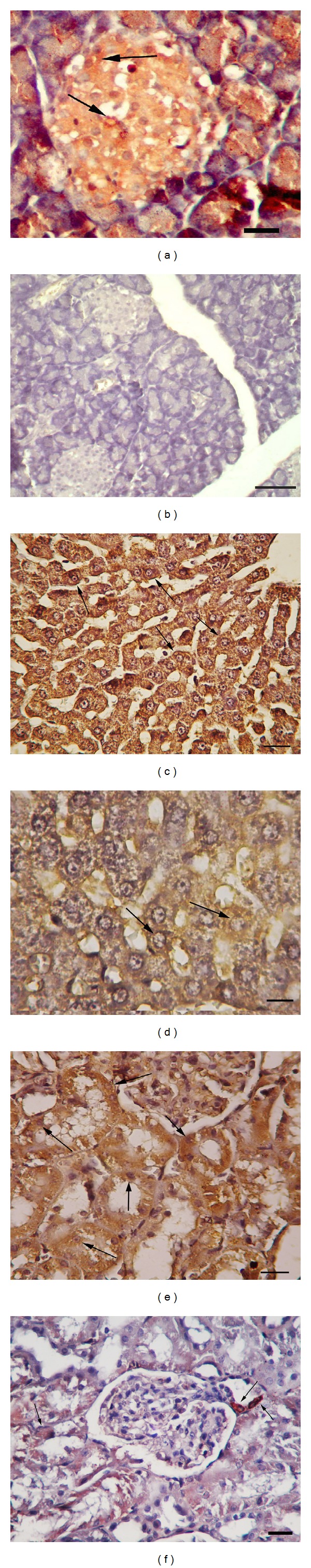
*COX-2  reactions.* (a) Severe positive immunoreaction in endocrine islets of pancreas in study group (arrows), Bar = 50 *μ*m. (b) No immunoreaction in Langerhans islets of pancreas in control group, Bar = 100 *μ*m. (c) Moderate immunoreaction in hepatocyte in study group (arrows), Bar = 100 *μ*m. (d) A few immunoreactions in liver (arrows) in control group, Bar = 50 *μ*m. (e) Strong immunopositive reaction in nonmacula densa area in kidney (arrows) in study group, Bar = 100 *μ*m. (f) A few immunoreactions in kidney tubul cells in macula densa (arrows) in control group, Bar = 100 *μ*m. ABC-P method with hematoxylin counterstaining was used for all tissues. The right column belongs to study group and left column is belong to control group.

**Figure 3:**
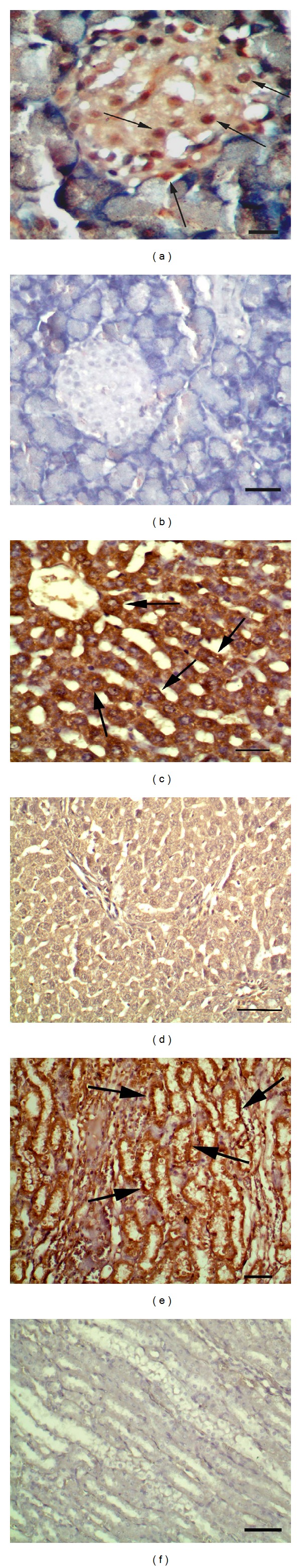
*CSR  reactions*. (a) Severe expression in nucleus and cytoplasms of endocrine islets of pancreas in study group (arrows), Bar = 50 *μ*m. (b) No immunoreaction in Langerhans islets of pancreas in control group, Bar = 100 *μ*m. (c) Severe immunopositive reaction in hepatocyte in study group (arrows), Bar = 100 *μ*m. (d) A few immunoreactions in liver (arrows) in control group, Bar = 100 *μ*m. (e) Strong immunopositive reaction in tubular epithelial cells in kidney (arrows) in study group, Bar = 200 *μ*m. (f) Very slight immunoreactions in kidney in control group, Bar = 200 *μ*m. ABC-P method with Hematoxyline counterstaining was used for all tissues. The right column belongs to study group and left column belongs to control group.

**Figure 4:**
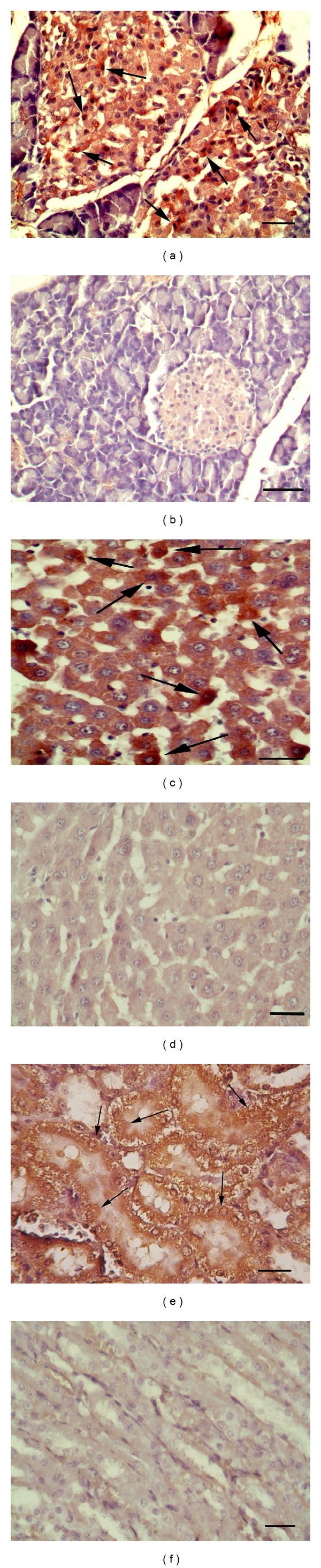
*HIF  reactions*. (a) Severe expression in endocrine islets of pancreas in study group (arrows), Bar = 100 *μ*m. (b) Slight immunoreaction in Langerhans islets of pancreas in control group, Bar = 100 *μ*m. (c) Severe immunopositive reaction in hepatocyte in study group (arrows), Bar = 50 *μ*m. (d) A few immunoreactions in liver in control group, Bar = 50 *μ*m. (e) Strong immunopositive reaction in tubular epithelial cells in kidney (arrows) in study group, Bar = 50 *μ*m. (f) Very slight immunoreactions in kidney in control group, Bar = 100 *μ*m. ABC-P method with Hematoxyline counterstaining was used for all tissues. The right column belongs to study group and left column belongs to control group.

**Figure 5:**
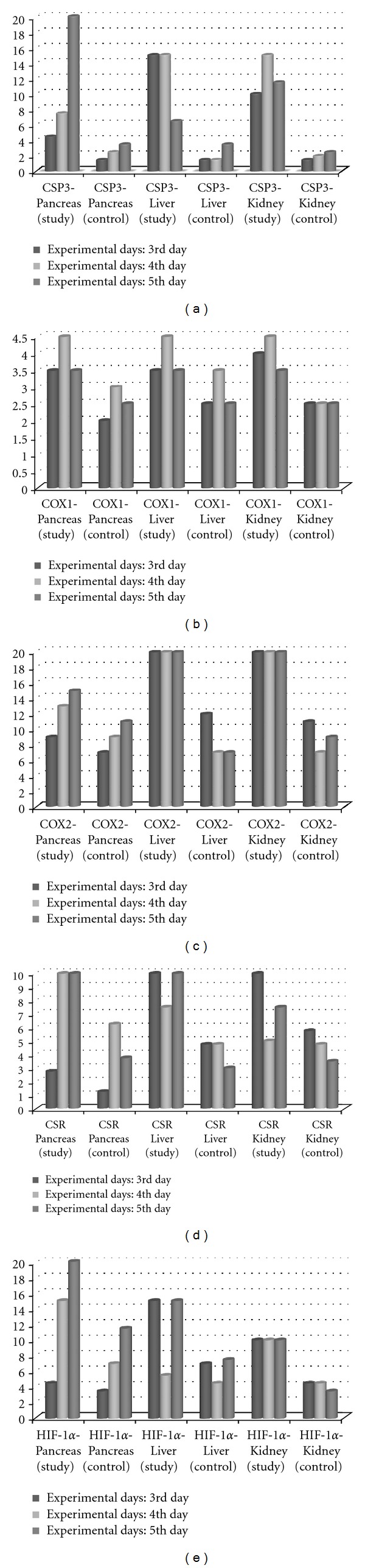
Statistical analysis results of (a) Caspase-3, (b) COX-1, (c) COX-2, (d) CSR, and (e) HIF-1*α*immunopositive cell numbers.

Hyperglycemia and ketonuria was initially observed in both rats in study group 3 days after administration. There were no glucosuria and ketonuria in the control group. Biochemical results of blood and urine were shown in Table 1. No macroscopical changes were observed in organs in both groups. At the histopathological examination of pancreas, degenerative and necrotic beta cells were seen in Langerhans islets in study group. At microscopical examination slight degenerative changes were observed in liver and tubular epithelial cells of the kidney. Immunohistochemical observation of caspase-3, COX-1, COX-2, CSR, and HIF-1*α* immunostained sections revealed severe damage in these organs in early stages of the DM. Statistical results of immunohistochemical observation were shown in graphic. Caspase-3 immunopositive cell numbers were markedly increased in pancreatic islets in study group. In addition to pancreas, caspase-3 immunopositive reaction was higher in liver hepatocyte in study group than controls and strong immunoreactions were observed in kidney tubular epithelial cells (Figures 1(a), 1(b), 1(c), 1(d), 1(e), and 1(f)). Slight increases in COX-1 reaction were observed in pancreas, liver, and kidney in study group's rats. In kidneys, immunohistochemical examination revealed that the expression of COX-1 localized on collecting tubules. COX-2 immunoreactive cells were markedly increased in study rats compared with controls in all examined organs. In control group, COX-2 positive immunostaining was observed in individual kidney tubular epithelial cells. Marked immunopositivity was demonstrated in macula densa and nonmacula densa tubules of kidney in study group (Figures 2(a), 2(b), 2(c), 2(d), 2(e), and 2(f)). In both groups, CSR immunopositive immunoreactions were noticed in cytoplasm of cells in the organs. But reaction was prominent in study group. Immunopositive reaction was also observed in nucleus of the some cells in Langerhans islets of pancreas. Similar CSR reaction was noticed in hepatocytes and both proximal and distal tubular epithelial cells of the kidney (Figures 3(a), 3(b), 3(c), 3(d), 3(e), and 3(f)). HIF-1*α* immunoreactions markedly increased in the study group while the controls were negative. Langerhans islets of pancreas exhibited markedly HIF-1*α* immunopositive reactions. Slight immunoreaction was detected in hepatocytes of the liver. Strong HIF-1*α* reaction was observed in both proximal and distal tubular epithelial cells of the kidney in study group (Figures 4(a), 4(b), 4(c), 4(d), 4(e), and 4(f)). Positive immunoreactivity was noted by an intense brown color (DAB). All of the markers were gradually increased related to days from induction of DM in this study (Figures 5(a), 5(b), 5(c), 5(d), and 5(e)).

## 4. Discussion

STZ administration to mature rats induces severe and permanent diabetes, with a decrease in insulin levels, to produce a cytotoxic model of diabetes very similar to type I DM. Streptozocin damages *β* cells of the islets of Langerhans in the pancreas [16]. Streptozotocin-induced diabetes in the rats is being employed extensively for studies into the immunopathogenesis of DM [17]. Although several studies have examined the underlying immune cellular and molecular changes during disease in this model, investigations on the early stages and cell injury in different organs have been limited. Cell damage is the main reason of necrosis and numerous agents can cause this process. The main reason of cellular injury is hypoxia and it is commonly seen. Calcium is main player of the cell damage and activates both plasma membrane and mitochondrial injuries which are cell damage pathways. COX enzymes play a major role in cellular damage process. Cell death is the last stage of the cellular damage and it can occur by apoptosis or necrosis. Caspase-3 is the main marker of the apoptosis [18]. This study planned to examine the role of calcium, hypoxia, and apoptosis in early stages of DM in different cells by using HIF-1*α*, COX-1 and -2, CSR and Caspase-3 by immunohistochemical method.

DM results from the progressive destruction of beta cells of Langerhans islets [19]. Multiple mechanisms have been proposed as effectors of beta cell destruction [20, 21]. Although DM is a chronic and progressive disease, initial lesions can be seen in very early stages. Previous studies reported that initial lesions occur after 3 days of DM induction in rats [22, 23]. Similar findings were observed in this study, and glucosuria was detected three days after streptozotocin treatment. Tissue sections of pancreas, liver, and kidney were examined histopathologically and immunohistochemically at 3rd, 4th, and 5th days. This study demonstrated that increased expression of caspase-3, COX-1 and -2, CSR, and HIF-1*α* in islet of Langerhans, liver, and kidney in streptozotocin induced DM in rats. These results supported the idea that cellular damages due to DM can occur in very early stages of the disease. Our results showed that different mechanisms may play role in diabetes like as hypoxia, apoptosis, and calcium influx in degenerative changes in cells.

Caspases are cysteine-aspartyl specific proteases that play a key role in apoptosis [24]. Caspase-3 is one of the effector caspases downstream of apoptotic pathways. Gene targeting strategies have provided valuable tools to study the physiologic function of individual caspases in vivo and have shown their roles not only in apoptosis but also in other fundamental cellular processes [4]. Several in vitro studies have suggested that caspase dependent apoptotic pathways are essential for *β* cell apoptosis [25]. The underlying mechanism of tubular changes in kidney in diabetes, however, is unclear. One attractive mechanism is apoptosis, which has been demonstrated to mediate cell death in a variety of renal diseases, including diabetic nephropathy [26]. Indeed, apoptosis was detected in renal proximal tubular cells of different species including experimental animals and patient with diabetes, suggesting that tubular apoptosis may precede tubular atrophy in diabetes [27, 28]. In this study, apoptotic activity in pancreatic islets was also observed in the study group's rats and was an agreement with previous studies. At the same time we also observed increased apoptotic activity in the hepatocytes and kidney tubular cells. This study showed that possible mechanism of occurrence of liver and kidney problem in diabetes may be related to the increased apoptotic activity.

Many studies using streptozotocin-induced type 1 diabetic rats have shown an increase in COX-2 production in kidney [23]. Therefore, in the present study, the kidneys of study and control rats were investigated histologically and immunohistochemically, and changes in the renal expression of COX-2 during the DM induction were marked in the study group compared to control group. Marked increase in COX-2 expressions was observed in the renal proximal tubules and macula densa in the study group as compared with the control group. Only slight increases were observed in COX-1 expression in the study group. These results were corresponding to previous observations [23]. The possible cause of the increased COX-2 protein expression in kidneys of study group rats may be related to that the tissue changes also have a pathophysiological impact in modulating renal hemodynamics in diabetes.

Calcium is known to be an important intracellular messenger. Ca^2+^ plays a key role in numerous cellular processes, such as maintaining membrane potential and controlling hormonal secretion and cellular proliferation and differentiation [8]. The mechanisms governing extracellular calcium homeostasis maintain its near constancy to ensure continual availability of calcium ions for their multiple intra- and extracellular functions. CSR expression has an important role in many physiological situations. It is involved in calcium metabolism regulation in many cells such as parathyroid [29], bone [30], kidney cells [31], fibroblasts [32], antral gastrin cells [33], epithelial cells [34], oligodendrocytes [30], renal cells, retina, osteoclasts and osteoblasts, vessels smooth muscle cells, and on some brain cells [8]. In this study CSR expression was demonstrated in pancreas, liver, and kidney in diabetes-induced rats. Expression of the CSR was more prominent in the study group than the control group. Marked increase was observed in both nucleus and cytoplasm of the cells of Langerhans islets of pancreas in the study group. Little expression was seen in the control group and only in cytoplasm. Strong immunopositive reaction was observed in collective tubules of kidney compared to the controls. Hepatocytes also expressed CSR in both groups, and reaction was more prominent in the study group.

Hypoxia is the main regulator of HIF-1*α* expression and function in some conditions such as diabetes pathogenesis [35, 36]. HIF-1*α* is one of the important members of the bHLH-PAS family [37] and functions as an obligate dimer with other family members, including aryl hydrocarbon receptor (AhR) nuclear translocator (ARNT) [38]. HIF-1*α* degradation occurred by HIF prolyl hydroxylases. HIF-1*α* degradation can be considered cellular oxygen sensors, because their activity varies in the range of physiologic/pathologic oxygen tensions [39]. It is very important to detect HIF activation in tissue sections by immunohistochemical methods in detection of nuclear HIF-1*α* [40]. HIF activity can be modulated by a number of factors such as hydrogen peroxide and superoxide [41]. Diabetes can cause increased production of reactive oxygen species [42]. In the renal medulla, NAD(P)H oxidase activity can cause increased superoxide in the thick ascending limbs of the loop of Henle [43]. Superoxide may both intensify renal medullary hypoxia and reduce hypoxia adaptation in diabetes [44]. In this study, although HIF-1*α*-negative in the control group's pancreas, increase reaction was seen in the liver and kidney in the study group. The most marked reaction of HIF-1*α* was observed in Langerhans islets of the pancreas.

## 5. Conclusion

As a result, this study showed that marked immunoreaction can be seen in the pancreas, liver and kidney with caspase-3, COX-1, COX-2, CSR, and HIF-1*α* in diabetes-induced rats. These reactions became prominent related to days after induction. The possible cause of the increase may be related to cellular damage by different routes. Increase of severity of the immunoreactions related to days also supported this idea. The most marked reaction was observed in Langerhans islets of pancreas with all of the markers in this study. But liver, and kidney can be affected in very early stages of the disease. These results indicated that cellular damage in DM showed both hypoxia and calcium influx in the cells. Apoptosis can cause marked lesions in organs. This study showed that although diabetes is a chronic disease, its affects can be seen in cells in early stages.

## Figures and Tables

**Table 1:** Blood and urine values of the rats in groups.

Study group	Blood glucose (mmol/L)	Ketonuria
3rd day	10.15 ± 2.18	—
4th day	11.84 ± 1.84	+
5th day	9.91 ± 1.69	+

Control group		
3rd day	4.05 ± 1.42	—
4th day	5.67 ± 0.94	—
5th day	6.07 ± 0.86	—

Reference values	2.28–7.50	—

The differences between the means of groups are statistically significant (*P* < 0.05).
